# Perfusion of the skin’s microcirculation after cold‐water immersion (10°C) and partial‐body cryotherapy (−135°C)

**DOI:** 10.1111/srt.12703

**Published:** 2019-04-30

**Authors:** Erich Hohenauer, Tom Deliens, Peter Clarys, Ron Clijsen

**Affiliations:** ^1^ Department of Business Economics, Health and Social Care University of Applied Sciences and Arts of Southern Switzerland Landquart Switzerland; ^2^ International University of Applied Sciences THIM Landquart Switzerland; ^3^ Department of Movement and Sport Sciences, Faculty of Physical Education and Physiotherapy Vrije Universiteit Brussel Brussels Belgium

**Keywords:** blood flow, cryotherapy, laser speckle contrast imaging, perfusion

## Abstract

**Background:**

Investigations of the perfusion of the skin's microcirculation with laser speckle contrast imaging (LSCI) after cold treatments are rare. Therefore, the aim of this study was to compare the effects between cold‐water immersion (CWI) conduction and partial‐body cryotherapy (PBC) convection on perfusion of the microcirculation and skin temperature on the thigh.

**Materials and Methods:**

Twenty healthy males were randomly allocated to CWI (10°C for 10 minutes) or PBC (−60°C for 30 seconds, −135°C for 2 minutes). Perfusion and skin temperature measurements were conducted on the anterior thigh region up to 60 minutes post‐treatment.

**Results:**

Cold‐water immersion decreased perfusion of the microcirculation significantly compared to baseline values between 10 minutes (*P* = 0.003) and 30 minutes (*P* = 0.01) post‐treatment. PBC increased perfusion of the microcirculation and decreased skin temperature only at the first measurement interval (0 minute, both *P* = 0.01) post‐treatment. Additionally, local skin temperature was significantly decreased compared to baseline values only after CWI up to 30 minutes (*P* = 0.04) post‐treatment.

**Conclusion:**

Cold‐water immersion reduced local skin microcirculation and skin temperature while PBC only slightly increased the perfusion of the microcirculation immediately after the treatment. For cooling purposes, the conduction method seems superior compared to the convection method, assessed with a LSCI device.

## 1 INTRODUCTION

The use of external cold treatments to extract heat from the body has a long history in rehabilitation sciences.^1^ Local treatments, with cold packs and ice sprays, are established on‐field strategies to relieve pain although its sustainability is discussed controversially in the literature.^2^ The effectiveness of cold treatments to relieve pain can be attributed primarily to its supposed effects on decreasing cell metabolism and reduction in nerve conduction velocity.^3^, ^4^ Beside local treatments, cold treatments that cover large skin surface areas were introduced to treat even systemic pain situations like rheumatoid arthritis, multiple sclerosis, or arterial hypertension.^5^, ^6^, ^7^ Cold‐water immersion (CWI) is a popular technique, and its effectiveness to relieve pain has been studied extensively in earlier published studies with controversial results.^8^, ^9^ Another way of extracting heat from the body is the use of partial‐body cryotherapy (PBC). During this treatment, participants stand in upright position in a cryocabin and cold air is produced through vaporization of liquid nitrogen. The treatment temperature of the vaporized air can reach up to −195°C with exposure times of up to 3 minutes.^10^

Assessing skin microcirculation has become an easily accessible and representative indicator to understand microvascular function.^11^ The observation of microvascular function has been highlighted to be of clinical importance as it plays a crucial role in physiological processes of tissue oxygenation and nutritional exchange.^12^ It has been demonstrated that adequate tissue oxygen delivery is dependent on microcirculatory functioning, and thus, maintained tissue perfusion is a key indicator of injury and disease.^13^ The pathophysiology of the skins’ circulatory behavior has been studied in many popular fields such as peripheral artery diseases,^14^ hypertension,^15^ type 2 diabetes,^16^ and primary aging.^17^

Laser Doppler flowmetry (LDF) and laser Doppler perfusion imaging (LDPI) are commonly used, non‐invasive methods to measure skin microcirculation.^11^, ^18^ Another established technology for assessing skin microcirculation is Tissue Viability (TiVi).^19^, ^20^ Laser speckle contrast imaging (LSCI) is a relatively new promising non‐contact technology for assessing microcirculation of the skin.^21^ A speckle pattern is an interference pattern produced by light reflected or scattered from different parts of the illuminated surface.^22^

It is well known that cold applications lead initially to a strong vasoconstrictive reaction of the skin which contributes to the cold‐induced analgesic effect.^4^, ^23^ Although this response is primarily triggered from local control systems, the sympathetic vasoconstrictor system is also involved in these processes.^24^ The release of norepinephrine and other co‐transmitters seem to contribute to the cold‐induced vasoconstriction.^25^, ^26^ The narrowing of blood vessels is an effective way of the body to minimize heat loss although the extraction of heat is the primary mechanism of cold treatments to facilitate a therapeutically or medical effect.^27^, ^28^, ^29^ Cutaneous vasoconstriction has been demonstrated to reduce skin temperature,^30^ skin blood flow,^31^ and nerve conduction velocity^3^ and thus leading to pain relief.^4^, ^32^

Although cold water and cold air treatments are popular and competing pain‐relieving strategies, to our knowledge, no direct comparison between CWI and PBC on the perfusion of the microcirculation of the skin has been published, using a LSCI device. Therefore, the aim of this study was to describe and compare the behavior of the perfusion of the microcirculation of the skin and the local skin temperature after CWI and PBC during a 60‐minute follow‐up period.

## 2 MATERIALS AND METHODS

### 2.1 Participants and design

In this randomized trial, a total of n = 20 healthy Caucasian males (age: 26.7 ± 3.9 years, height: 177.4 ± 8.7 cm, weight: 76.1 ± 6.2 kg) voluntarily participated. All participants were non‐smokers and were regularly involved in physical endurance training (running and cycling). All participants were screened for eligibility and excluded in case of skin abnormalities (eg, scars on the measurement site and psoriatic skin), or in case, they were allergic to cold (including Raynaud's disease), had cardiovascular diseases, or took any medication. On the day of the measurements, the participants were randomly allocated (by drawing lots) into either the CWI (n = 10) or the PBC group (n = 10). The participant's characteristics can be observed in Table 1. Participants were informed about the study content and protocol which was approved by the local ethical committee of Zurich (PB_2016‐01125) in accordance with the Declaration of Helsinki (ICH‐GCP).

**Table 1:** Characteristics of the sample (mean ± SD)

Parameters	CWI (n = 10)	PBC (n = 10)	*P*‐value
Age (y)	26.8 ± 3.7	26.6 ± 4.2	0.90
Height (cm)	173.6 ± 7.2	180.9 ± 8.8	0.06
Mass (kg)	76.4 ± 6.8	75.8 ± 5.8	0.07
BSA (m^2^)	1.90 ± 0.06	1.96 ± 0.08	0.06
Body fat (%)	16.5 ± 4.6	21.0 ± 7.2	0.12
∑ 9 SF (cm)	84.8 ± 31.6	94.3 ± 31.6	0.51
Thigh SF (cm)	12.1 ± 6.6	12.9 ± 6.3	0.78

Abbreviations: BSA, body surface area; SF, skinfold.

### 2.2 Experimental design

On the day of the experiment, participants were acclimatized for the duration of 20 minutes to the laboratory conditions of 21 ± 1°C and relative humidity of 40 ± 5%. During this period, participants were in supine position, wearing only swimming trunks. A region of interest (ROI) of 21 cm^2^, measured from the anterior patellar base in proximal direction, was clearly marked during the acclimatization period, to obtain valid microcirculation results of the left anterior thigh. Additionally, a single thermochron was taped (3M, Tegaderm) on the mid‐section of the right anterior thigh to observe the effects on the local skin temperature. After the acclimatization period, the baseline measurements were conducted. After the baseline measurements, the participants were either immersed in cold water or entered the cryocabin. The follow‐up measurements were performed after the treatments (0 minute) and in 10‐minute intervals up to 60 minutes post‐treatment.

### 2.3 Measurement of the perfusion of the microcirculation of the skin

Skin perfusion was measured with a LSCI system (moorFLPI2, Moor instruments) working with a wavelength of 750 nm in supine position. Calibration of the system was successfully performed 1 day prior to the experiment. To minimize the risk of confounding factors, daylight and other sources of light were diminished as well as movements of the device during the measurements. The participants were instructed to breathe normally and not to talk during the measurements. To obtain reliable data, the distance between the measured skin area and the LSCI device was controlled with the aiming laser function. These two lasers converge into a single point at a distance of 25 cm between the surface and the LSCI device. To receive high‐resolution images (752 × 580 pixels), the temporal filter was set to 25 frames (1 s/frame) with an interval of 5 seconds.

### 2.4 Measurement of the local skin temperature

Skin temperature of the thigh was measured with the iButton system (Maxim Integrated) in supine position. The thermochron (DSL1922L) was placed on the mid‐section of the right frontal thigh (midway between the proximal patella and the inguinal crease) to obtain continuous information about the local skin temperature. The iButton data logger system can be used to obtain a valid measurement of human skin temperature.^33^

### 2.5 Cold treatments

After the acclimatization and baseline measurements were conducted, the participants received one of the two possible cold treatments in a randomized order.

During the CWI, participants were immersed up to the sternal level in cold water (10 ± 1°C). The water temperature was constantly monitored by using a multimeter device (Voltacraft MT52), and crushed ice was added if necessary. Participants sat on a stool with the hands on the edge of the water tank. After the 10‐minute immersion time, participants left the tank, were patted dry, and laid down in supine position for the 60‐minute follow‐up measurements.

During PBC, participants entered a mobile cryocabin (Cryomed sro, Cryosauna Space Cabin) and were exposed to vaporized liquid nitrogen for the total duration of 2.5 minutes. The treatment was performed with following specifications: 30‐second exposure at −60°C and 2‐minute exposure at −135°C. This experimental setup was already used in the previously published studies.^34^, ^35^, ^36^ During this treatment, participants wore woolen boots, as recommended by the manufacturer. Participants were instructed to place the hands at the edge of the cabin and to slowly turn around the *y*‐axis. After the 2.5 minutes lasting treatment, participants laid down in supine position for the 60‐minute follow‐up measurements.

### 2.6 Statistical analysis

All data were checked for normality using the Shapiro‐Wilk test, allowing to perform parametric statistics. Normalized values (% ± SD) to baseline values were used for the analysis of the perfusion of the skin's microcirculation while continuous data were used for the analysis of the local skin temperature. Repeated measures ANOVAs mixed design were used to investigate effects of time (Baseline, 0, 10, 20, 30, 40, 50, 60 minutes) and condition (CWI vs PBC), for both the outcome measures microcirculation and skin temperature. Independent samples *t* tests were used to observe between‐group differences.

Post hoc pairwise comparisons with Bonferroni correction were used to assess within‐group differences. The observed power is expressed as 1−*ß*, and the significance level was set at *α* = 0.05. All statistical analyses were performed in SPSS (SPSS Inc) version 24.0.

## 3 RESULTS

**Figure 1:**
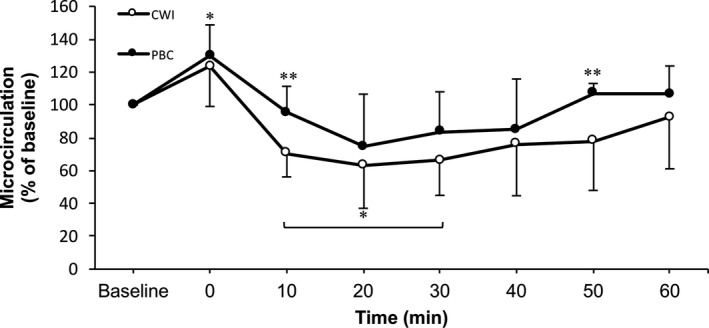
Perfusion of the microcirculation of the left anterior thigh in function of time. Values are normalized to baseline (% mean ± SD) with respect to their initial values. *P < 0.05 within‐group difference compared to baseline. **P < 0.05 between CWI and PBC

A significant time (*F*_[7,12]_ = 22.04, *P* < 0.001, 1−*ß* = 1.0) with no condition (*F*_[1,18]_ = 3.67, *P* = 0.07 1−*ß* = 0.44) but significant time × condition interaction (*F*_[7,12]_ = 6.66, *P* = 0.002, 1−*ß* = 0.98) was observed for perfusion of the skin's microcirculation. Pairwise comparisons revealed significant decreased values in the perfusion of the skin's microcirculation compared to baseline in the CWI group at 10 minutes (*P* = 0.003), 20 minutes (*P* = 0.04), and 30 minutes (*P* = 0.01). In the PBC group, there was only a significant increase in the perfusion of the skin's microcirculation compared to baseline immediately after the treatment (0 minute; *P* = 0.01). The independent samples *t* test indicated significant differences in the perfusion of the skin's microcirculation between CWI and PBC at 10 minutes (*t*[18] = −3.67, *P* = 0.002) and 50 minutes (*t*[18] = −2.95, *P* = 0.008), with CWI showing lower values than PBC at these time points. These differences can be observed in Figure 1.

**Figure 2:**
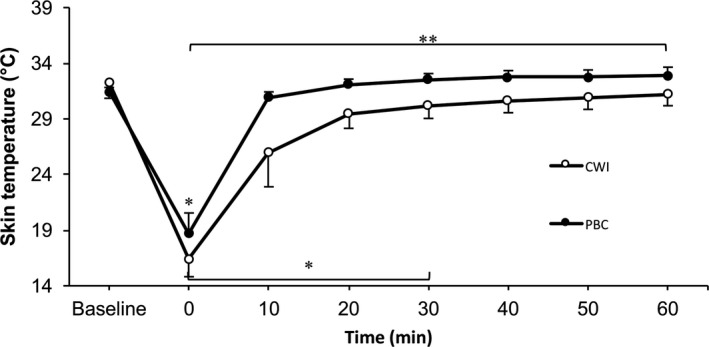
Skin temperature of the right anterior thigh in function of time. Values are means ± SD. *P < 0.05 within‐group decrease compared to baseline. **P < 0.05 between CWI and PBC

A significant time (*F*_[7,12]_ = 132.08, *P* < 0.001, 1−*ß* = 1.0), condition (*F*_[1,18]_ = 38.30, *P* < 0.001, 1−*ß* = 1.0), and time*condition interaction (*F*_[7,12]_ = 5.93, *P* = 0.004, 1−*ß* = 0.96) were observed for the local skin temperature. Significantly decreased within‐group values for skin temperature compared to baseline were observed in the CWI group at 0 minute (*P* < 0.001), 10 minutes (*P* = 0.01), 20 minutes (*P* = 0.01), and 30 minutes (*P* = 0.04) after the treatment. Within the PBC group, a significant decrease in skin temperature compared to baseline was observed post‐treatment (0 minute; *P* < 0.001). Significant between‐group differences in skin temperature were observed through the entire follow‐up period at 0 minute (*t*[18] = −2.88, *P* = 0.01), 10 minutes (*t*[18] = −4.98, *P* < 0.001), 20 minutes (*t*[18] = −5.73, *P* < 0.001), 30 minutes (*t*[18] = −5.64, *P* < 0.001), 40 minutes (*t*[18] = −5.42, *P* < 0.001), 50 minutes (*t*[18] = −4.76, *P* < 0.001), and 60 minutes (*t*[18] = −4.35, *P* < 0.001), with CWI showing consistently lower values than PBC across these time points. All differences regarding skin temperature can be observed in Figure 2.

## 4 DISCUSSION

The aim of this study was to describe and compare the behavior of the perfusion of the microcirculation of the skin and the local skin temperature after CWI and PBC during a 60‐minute follow‐up period. The primary findings of this study are that CWI led to cold‐induced vasoconstriction which was not observed after PBC (Figure 1). The results of the current study are partially in line with those obtained from Mawhinney et al.^37^ These authors observed significantly reduced cutaneous vascular conductance (CVC) of the thigh after both cold water and cold air compared to baseline values. Although both cold treatments led to vasoconstriction of the thigh's skin up to 40 minutes post‐treatment, CWI reduced the perfusion of the microcirculation of the skin to a higher extent (CWI: by ~75%, WBC: by ~55%, *P* < 0.001) compared to the WBC treatment. The current study results indicate that cold water reduces the skin's perfusion of the microcirculation significantly up to 30 minutes post‐treatment. In contrast with the findings from Mawhinney et al, we did not observe significantly circulatory reductions after the use of cold air. Mawhinney et al used a whole‐body chamber system that cooled the entire body surface (30 seconds at −60°C, 2 minutes at −110°C), while the participants’ bodies in the present study were only partially cooled (head and hands out of the cabin). Additionally, Mawhinney et al assessed cutaneous blood flow with a non‐contactless LDF and not with a contactless LSCI, as used in the present study. Compared to traditional LDF measurements, LSCI allows measurements of fast changes in skin blood flow over wide skin areas with very good inter‐day reproducibility due to good temporal and spatial resolutions.^38^, ^39^, ^40^ Consequently, LSCI technology can investigate wide skin areas and fast changes of skin microcirculation compared to LDF devices.^38^, ^40^

Similarly to the results from Mawhinney et al are those obtained from another research group by Costello et al.^41^ These authors demonstrated that the red blood cell concentration was also decreased after both cold water and cold air. Furthermore, these authors observed that the red blood cell concentration started to stabilize after approximately 35 minutes. Similarly, the current study results demonstrate that the microcirculatory values start to return to baseline values between 30 and 40 minutes after the CWI treatment. However, it should be considered that the cryotherapy treatment in the study of Costello et al was a whole‐body chamber (20 seconds at −60°C, 3 minutes at −110°C) and not a PBC treatment (30 seconds at −60°C, 2 minutes at −135°C), as used in the current study. Furthermore, Costello et al assessed red blood cell concentration of the skin with a high‐resolution TiVi camera, which is different compared to the speckle laser camera system, used in the current study.

Tissue Viability is a welcome tool in the study of microcirculation but the depth of measurement for TiVi is slightly higher compared to the LSCI measurement, where the superficial measurement corresponds to the nutritional supply.^42^

Beside the different approaches to assess the cutaneous microcirculatory properties, the differences in cooling methods might have affected the results, as described elsewhere.^34^, ^43^

In the present study, local skin temperature of the thigh was measured with a conductive thermo‐button system, which demonstrated significantly decreased values after CWI treatment up to 30 minutes post‐treatment. The PBC values were only decreased after the treatment (0 minute) but were significantly different from the CWI values throughout the entire follow‐up period. Our findings are in line with those obtained from other researchers who observed that CWI reduced skin temperature on the thigh/knee to a larger amount compared to WBC, observed between 40‐ and 60‐minute follow‐up period.^30^, ^37^, ^44^ These authors assessed skin temperature with a skin thermistor and contactless infrared thermal imaging techniques. Beside the evaluation techniques, a possible explanation for the differences between the studies might be the thermal conductivity, which differs between cold water and cold air. The thermal conductivity is 24 times higher for water (0.58 W/[m × K]) compared to air (0.024 W/[m × K]),^32^ which might explain the greater reduction of skin temperature in the CWI group compared to WBC and PBC treatments, respectively.

## 5 CONCLUSION

The results of the current study show that CWI decreases local perfusion of the microcirculation of the skin to a greater extent than a PBC treatment, measured with a contactless LSCI. This study suggests that the use of skin contact‐free lasers provides similar results to traditional methods with skin contact, in the research field of cryotherapy.
